# Overwintering Does Not Affect Microbiota Diversity in *Halyomorpha halys* : Implications for Its Ecology and Management

**DOI:** 10.1111/1758-2229.70116

**Published:** 2025-06-10

**Authors:** Riccardo Piccinno, Giulio Galla, Gerardo Roselli, Mirco Rodeghiero, Valerio Mazzoni, Lloyd Stringer, Heidi Christine Hauffe, Gianfranco Anfora, Omar Rota‐Stabelli

**Affiliations:** ^1^ Center Agriculture Food Environment (C3A) University of Trento Trento Italy; ^2^ Department of Biology and Biotechnology “Lazzaro Spallanzani” University of Pavia Pavia Italy; ^3^ Conservation Genomics Research Unit Research and Innovation Centre, Fondazione Edmund Mach Trento Italy; ^4^ Forest Ecology Unit Research and Innovation Centre, Fondazione Edmund Mach Trento Italy; ^5^ Plant Protection Unit Research and Innovation Centre, Fondazione Edmund Mach Trento Italy; ^6^ The New Zealand Institute for Plant and Food Research Lincoln New Zealand; ^7^ Better Border Biosecurity (B3) Lincoln New Zealand; ^8^ National Biodiversity Future Center (NBFC) Palermo Italy

**Keywords:** 16S rRNA gene, agricultural pest, amplicon sequencing, brown marmorated stink bug, diapause, invasive species, metataxonomy, seasonal dynamics

## Abstract

Host‐associated microbial communities play an important role in regulating many aspects of insect biology, but changes in this microbiota during diapause and overwintering are still largely unknown. *Halyomorpha halys* is an invasive agricultural pest characterised by a unique overwintering strategy where individuals aggregate and enter a state of dormancy, making it an excellent model to study the relationship between microbiota and diapause. We investigated the bacterial diversity of wild *H. halys* specimens before and after dormancy using 16S rRNA gene amplicon‐sequencing. We found that microbiota varies between geographically neighbouring sampling locations, but there were no significant differences in microbial diversity or composition between populations sampled before and after diapause, despite stressful overwintering conditions. Such stability may relate to the highly specific taxa that dominate the stinkbug‐associated microbial community. In addition, we did not detect any strong association of stink bugs with phytopathogens, but we found that two populations harboured *Nosema maddoxi*, a microsporidian pathogen of stink bugs. Our results are relevant to the assessment of accidental spillovers of microorganisms in newly invaded areas and to the implementation of the sterile insect technique based on mass trapping, irradiation, shipping and release after diapause of wild individuals.

## Graphical abstract

We challenged the assumption that overwintering significantly alters host microbiota composition, finding no differences between *Halyomorpha halys* populations sampled before and after overwintering. This microbiota stability underscores its role in the invasive pest's resilience and provides insights for ecological management strategies.

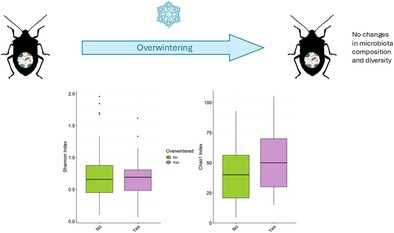

## 1 Introduction

Like all other multicelled species, insects establish strong associations with communities of microorganisms to support numerous processes, including nutrition (Bansal et al. 2014; Wang et al. 2024); pathogen resistance (Douglas 2015; Li et al. 2021); development (Chouaia et al. 2019; Morimoto et al. 2019; Zadra et al. 2023) and detoxification of plant metabolites acquired through diet (Douglas 2015; Coolen et al. 2024; Zhang et al. 2024). As shown for several species, part of the microbiota is often maternally inherited in insects (Duron and Hurst 2013; Shan et al. 2021). However, a number of environmental factors may directly or indirectly contribute to shaping insect‐associated microbiota (Moran et al. 2008). Indeed, in a wide number of insects, including pests, changes in microbial diversity are associated with diet and environmental parameters ( *Gryllus veletis* Alexander & Bigelow: Ferguson et al. 2018; *Spodoptera frugiperda* Smith: Jones et al. 2019; *Trichoplusia ni* Hübner: Leite‐Mondin et al. 2021; Chrysomelidae spp.: Magoga et al. 2023; *Drosophila suzukii* Matsumura: Martínez‐Solís et al. 2020; Mason et al. 2020; *D. melanogaster* Meigen: Sepulveda and Moeller 2020; *Apolygus lucorum* Meyer‐Dür: Guo et al. 2023; *Aphis gossypii* Glover: Wang et al. 2025; Pentatomomorpha spp.: Li et al. 2022). Most insects that are distributed in temperate regions employ overwintering strategies to ensure their population's survival throughout the winter (Vercher et al. 2023). During this period, individuals are exposed to harsh conditions, such as starvation and cold, which are also known to induce seasonal shifts in some insect microbial communities ( *Diploptera punctata* Eschscholtz: Ayayee et al. 2020; *G. veletis* : Ferguson et al. 2018; *Hermetia illucens* Yang et al. 2021).

The brown marmorated stink bug, *Halyomorpha halys* (Stål, 1855) (Hemiptera: Pentatomidae), is native to eastern Asia, but is considered an invasive species in the Middle East, Europe, the Americas and Africa (EPPO 2024). It was accidentally introduced to North America in the late 1990s (Hoebeke and Carter 2003) and to Europe in 2004 (Wermelinger et al. 2008; Musolin et al. 2022), likely as a result of international trade of goods (Valentin et al. 2017). This species has been steadily enlarging its distribution range into other temperate regions including Chile and Turkey (Bosco et al. 2018; Leskey and Nielsen 2018; Maistrello 2024; Maistrello et al. 2018). It feeds on more than 300 species of plants, including many crops (Tassini and Mifsud 2019), and it is a major agricultural pest in most of the countries it has successfully invaded (Leskey and Nielsen 2018). In Italy, the effect of *H. halys* on various crops has been severe (Bosco et al. 2018; Maistrello et al. 2017). *H. halys* was first described as a chill‐intolerant species (Cira et al. 2018), meaning that mortality is high when temperatures fall below 0^o^C (Cira et al. 2016; Denlinger and Lee Jr. 2010). However, in temperate conditions, adults minimise the risk of freezing by entering diapause in late autumn (Cira et al. 2018; Nielsen et al. 2017; Nielsen and Hamilton 2009; Watanabe et al. 1978), during which they stop feeding (Papa and Negri 2020). To improve their chances of survival during winter, *H. halys* aggregate in large groups in natural or artificial refuges, such as hollow trees, wooden sheds, cellars, or attics (Cira et al. 2016). In this species, diapause usually ends during spring in the Northern Hemisphere (Bergh et al. 2017). Depending on temperature, emergence from diapause can extend in some cases to late spring (Lowenstein and Walton 2018; Nielsen et al. 2017; Saulich and Musolin 2018). When overwintered adults emerge, they start feeding again on available host plants (Leskey and Nielsen 2018), and adults become reproductively active from the end of May (Nielsen and Hamilton 2009; Nielsen et al. 2017; Musolin et al. 2019; Reznik et al. 2022).

In recent years, the characterisation of *H. halys* midgut V4 tissues and egg samples using 16S rDNA amplicon sequencing has highlighted an obligate symbiotic association with bacterial species of the genus *Pantoea* across a wide range of geographic areas spanning the US west and east coasts (Bansal et al. 2014), suggesting that, as for other pest insect species, association with *Pantoea* spp. might be important for *H. halys* nutritional intake, development and growth (Bansal et al. 2014; Gonella et al. 2020; Gonella and Alma 2023). Further studies on *H. halys* have highlighted seasonal variation in microbial diversity (Fluch et al. 2024) and described the impact of the microsporidium *Nosema maddoxi* (Becnel et al.) on the survival, development and female fecundity (Preston et al. 2020b). Overwintered *H. halys* exhibit enhanced cold tolerance and reduced metabolic rates, allowing them to survive lower temperatures and prolonged periods without water. Diapausing individuals are better at conserving energy and water, which increases their survival rates in sheltered habitats compared to nonoverwintered individuals (Ciancio et al. 2021). It appears that the gut microbiota of *H. halys* before and after overwintering may reveal critical insights into how microbial communities influence the insect's survival, metabolism and overall fitness in response to seasonal environmental changes. Yet, we still know very little about the composition of microbiota associated with *H. halys* , its natural variation between geographically isolated populations, and the impact of overwintering on its microbiota, which might ultimately provide useful information for planning pest management strategies.

Here, we investigated the changes in the microbiota of *H. halys* collected in four populations before and after diapause in the Province of Trento (Italy) using high‐throughput amplicon sequencing on whole bugs to simultaneously profile the prokaryotic communities hosted by both internal tissues and the external surface of the host. We characterised the taxonomic composition and diversity of microbial communities for each population in relation to overwintering. Furthermore, to identify microorganisms that could potentially be transferred during release programmes using, for example, the sterile insect technique (SIT), our sequences were analysed for the presence of plant pathogens that could be vectored by *H. halys* . We detected the presence of *N. maddoxi*, a pathogenic microsporidian for various species of Pentatomids other than *H. halys* (Hajek et al. 2018) that, therefore, has the potential to be used as a biological control agent. We discuss how our results could be applied to enhance control strategies against this pest.

## 2 Materials and Methods

### 2.1 Sample Collection

**FIGURE 1:**
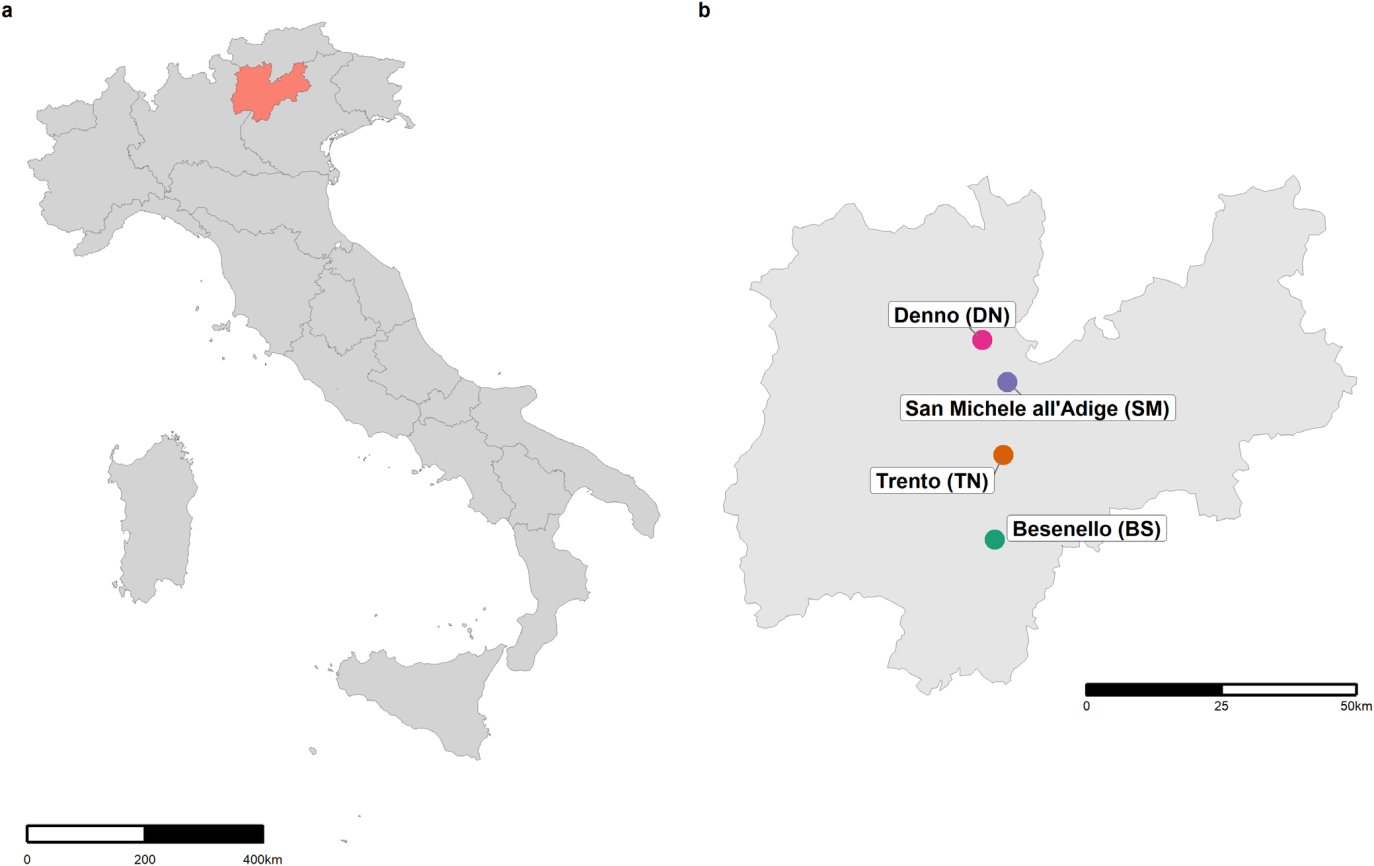
Map showing the four sampling locations for *Halyomorpha halys* in Italy (a) and the Province of Trento (b). Maps were generated using giscoR (Hernangómez 2023) to retrieve geographic boundary data directly from GISCO (Eurostat's geographic information system), ggplot2 (Wickham 2011; Wickham et al. 2016) as the foundational visualisation framework, ggspatial (Dunnington and Thorne 2020) to enhance spatial mapping capabilities (e.g., scale bars), and units (Pebesma et al. 2016) to manage unit conversions and scaling in geographic projections.

Individual *H. halys* adults were collected using sterile gloves and tweezers from single live traps baited with aggregation lures (Trécé, Adair, OK, USA; Suckling et al. 2019b) placed in each of four locations in the Province of Trento, Italy (Figure 1a): San Michele all'Adige (SM: 46°11′ 23″N, 11°08′17″E, altitude: 269 m a.s.l.); Denno (DN: 46°15′ 46″N, 11°03′42″E, 280 m a.s.l.); Trento (TN: 46°01′24″N, 11°08′00″E, 219 m a.s.l.) and Besenello (BS: 45°56′12″N, 11°06′39″E, 210 m a.s.l.) from October to November 2020 during the peak of aggregating behaviour. Immediately following collection, 10 individuals per site (40 bugs in total, hereafter ‘non‐overwintered’) were placed in sterile DNA/DNAse‐free 2 mL tubes and stored at −80°C until further processing. The remaining adults were reared in four cloth cages (30 × 30 × 30 cm; BugDorm, Taichung, Taiwan), one cage per sampling location. Following Roselli et al. (2023), the insects were initially placed in a greenhouse under natural photoperiod conditions at 15°C–18°C and a relative humidity of 70%, with *ad libitum* access to fresh tomatoes, green beans, carrots and apples (provided daily), and water provided on a wad of wet cotton, in preparation for overwintering. At the end of November, insects were transferred to an unheated wooden shed with unshuttered windows, that is, with ambient conditions similar to those chosen by wild *H. halys* for diapause. Cardboard rolls were put in the cloth cages to act as shelter. Temperature and humidity inside the shelter were monitored using a data logger EL‐USB‐2 (Lascar Electronics, Whiteparish, UK). Emerging overwintered adults (*n* = 21 in total: SM: 4, BS: 5, TN and DN: 6 each; hereafter: ‘overwintered’) were collected in March 2020 using sterile disposable gloves; each individual bug was placed in a sterile DNA/DNAse‐free 2 mL tube and stored at −80°C until DNA extraction.

### 2.2 DNA Extraction, Amplification and Sequencing

Under a BSL2 sterile hood (at the Plant Protection Unit of the Fondazione Edmund Mach, San Michele all'Adige, Trento, Italy), whole animals were placed in sterile 2 mL DNA/DNAse‐free tubes, flash frozen in liquid nitrogen for at least 30 s, then ground to powder using a Tissuelyser II (QIAGEN) set to 30 Hz for 1 min; 320 μL of enzymatic lysis buffer (Protocol: Pretreatment for Gram‐Positive Bacteria; Composition: 20 mM Tris‐Cl, pH 8.0; 2 mM sodium EDTA; 1.2% Triton X‐100) were added to each sample. This mixture was vortexed for 20 s and divided into two aliquots of approximately 160 μL each. One aliquot was used for DNA extraction while the other aliquot was frozen at −80°C for future use (archived at the Conservation Genomics Unit of the Fondazione Edmund Mach). We continued the DNA extraction by adding 18 μL of lysozyme (200 mg/mL) to one aliquot of *H. halys* homogenate. DNA extractions were carried out with the DNeasy Blood and Tissue kit (QIAGEN) by following manufacturer's instructions provided for the protocol ‘Pretreatment for Gram‐Positive Bacteria’ (full protocol provided in Supporting Information). Negative controls for DNA extraction (i.e., tubes containing all reagents but no bug) were included to detect kit or laboratory contamination. Quantification of DNA extracts as well as quality checks (i.e., 260/280 and 260/230 ratios) were performed using a Spark multimode microplate reader (Tecan, Switzerland) in UV/VIS mode. All DNA samples were diluted in DNA/DNAse/RNAse free water to a final concentration of 50 ng/μl.

For the amplicon sequencing analysis, the 16S rRNA gene region V3‐V4 was amplified using the KAPA HiFi HS ReadyMix (Roche). Specifically, PCR reactions were performed in a volume of 25 μL, containing 1X KAPA HiFi HS ReadyMix Buffer, the two primers 341F_ILL (5′‐TCGTCGGCAGCGTCAGATGTGTATAAGAGACAGCCTACGGGNGGCWGCAG‐3′) and 805R‐2_ILL (5′‐GTCTCGTGGGCTCGGAGATGTGTATAAGAGACAGGACTACNVGGGTWTCTAATCC‐3′) (Klindworth et al. 2013; Walters et al. 2015) to a final concentration of 0.3 μM each, dNTPs to a final concentration of 200 μM each and 100 ng of DNA (50 ng/μl). Amplification reactions were performed on a Veriti 96‐Well Fast Thermal Cycler (Applied Biosystems, USA) using the following parameters: an initial denaturation step at 95°C for 3 min, followed by 35 cycles consisting of 30 s at 95°C, 30 s at 55°C and 90 s at 72°C, and a final step at 72°C for 7 min. Negative controls for DNA amplifications (i.e., tubes containing all reagents but no sample DNA) were also included. Amplification success was estimated using the QIAxcel Advanced System (QIAGEN) with default parameters and reagents. Library preparation, quantification and sequencing were performed at the Sequencing and Genotyping Platform, Fondazione Edmund Mach using Illumina MiSeq 2x300bp with a minimum depth of 100,000 reads per sample.

Since the 16S rRNA gene region V3‐V4 of *Nosema maddoxi* cannot be amplified with the primers listed above, to screen for this taxon we amplified the microsporidian ribosomal SSU 16S rRNA gene using different primers (18f 5′‐CACCAGGTTGATTCTGCCTGAC‐3′, 1492r 5′‐GGTTACCTTGTTACGACTT‐3′), and the protocol provided by Hajek et al. (2018). Briefly, amplification reactions were performed in a volume of 20 μL, containing 1X Green GoTaq Flexi Buffer (Promega), 2 μM MgCl_2_, forward and reverse primers to a final concentration of 0.25 μM each, 2 U of GoTaq G2 Hot Start Taq Polymerase (Promega) and 100 ng of sample DNA (50 ng/μl). All reactions were performed on a Veriti 96‐Well Fast Thermal Cycler (Applied Biosystems, USA) using the following parameters: an initial denaturation step at 95°C for 3 min, followed by 40 cycles consisting of 30 s at 95°C, 30 s at 55°C and 90 s at 72°C and a final step at 72°C for 7 min. Negative controls for DNA amplifications were also included. Amplification success was estimated as detailed above. Microsporidian 18f‐1942r amplicons were then enzymatically purified using ExoI/FastAP (ThermoFisher Scientific) and sequenced using Sanger technology at the Sequencing and Genotyping Platform of Fondazione Edmund Mach to confirm amplification specificity.

### 2.3 Statistical and Bioinformatic Analyses

For the bacterial microbiota analysis, we used CutAdapt (Martin 2011) to remove adapters from the 16S rRNA V3‐V4 reads. The remaining analyses were performed in RStudio (Allaire 2012; R Core Team and Team 2022). DADA2 (Callahan et al. 2016) was used to filter the reads by quality, remove errors, merge the forward and reverse reads, remove chimeras and assign taxonomic names to the resulting ASVs using Silva v138 (Quast et al. 2013; Yilmaz et al. 2014). Decontam (Davis et al. 2018) was used to remove contaminant sequences considering the negative controls and using the prevalence method, and phyloseq (McMurdie and Holmes 2013) to compute microbial abundance, as well as alpha and beta diversity estimates. We used a Mann–Whitney U test (Fay and Proschan 2010; Mann and Whitney 1947) to evaluate differences in alpha diversity estimates between populations and between overwintering and prediapause individuals. We used the nonmetric multidimensional scaling (NMDS) approach in vegan to perform ordination of samples based on beta diversity estimates (Oksanen et al. 2018). PERMANOVA and ANOSIM were calculated using vegan (Oksanen et al. 2018); and pairwise adonis was calculated using pairwaise.adonis2 from pairwiseAdonis (Martinez Arbizu 2020) to test differences in beta diversity estimates between populations and seasons (overwintered vs. not overwintered). Mantel tests (Mantel 1967) were used to investigate population connectivity in relation to the similarity/diversity of microbiota profiles. Population‐level effect sizes of overwintering on alpha and beta diversity estimates were calculated using Cliff's delta (Cliff 1993), with the effsize R package (Torchiano 2020). Finally, DESeq2 (Love et al. 2014) was used to identify amplicon sequencing variants (ASVs) that were differentially abundant in overwintered and not overwintered bugs.

Microsporidian sequences were visualised using Chromas software (version 2.6.6). Forward and reverse reads were assembled using a Biopython script (Cock et al. 2009). Species assignment was performed using NCBI BLAST (Johnson et al. 2008) and the assembled sequences. We used R version 4.1.2 software (R Core Team and Team 2022) to compute standard statistics, and the ggplot2 R package (Villanueva and Chen 2019; Wickham 2011; Wickham et al. 2016) to draw graphs.

We additionally screened the taxonomic classification of amplified ASVs to confirm the presence of phytopathogens from the following genera: *Agrobacterium, Burkholderia, Erwinia, Pectobacterium, Pseudomonas, Candidatus Phytoplasma, Ralstonia* and *Xanthomonas*.

## 3 Results

The 16S rDNA amplicon sequencing of 61 *H. halys* individuals generated a median number of 88,853 sequence reads per library (ranging from 46,234 to 158,761 reads), resulting in the assembly of 2551 ASVs. By comparing the ASVs found in the negative controls with the dataset, we were able to identify and remove 28 ASVs that were potential contaminants (14,380 reads in total): this resulted in a final dataset consisting of 2523 ASVs.

### 3.1 Microbial Composition and Diversity Among Populations in Relation to Overwintering

**FIGURE 2:**
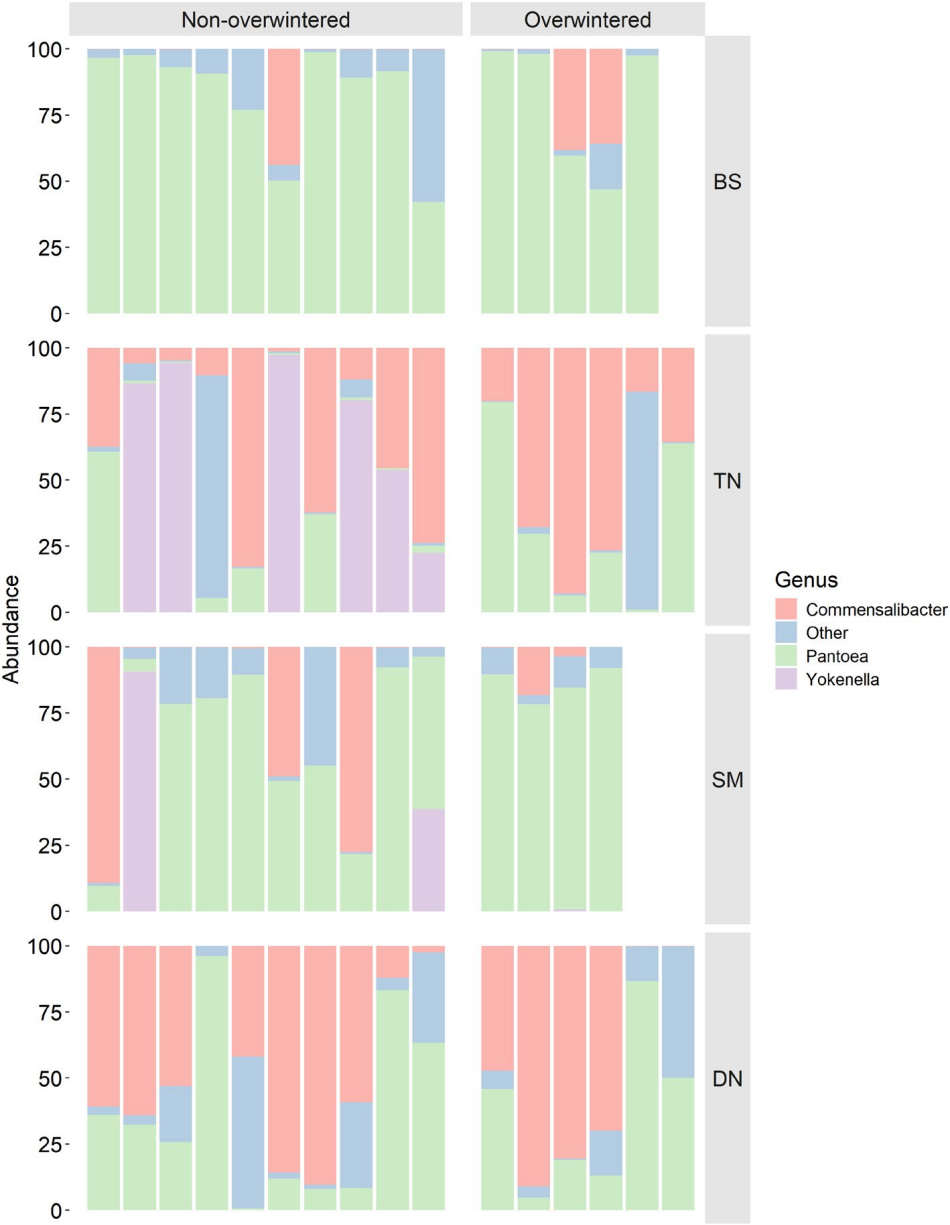
Abundance plots computed considering bacterial genera in all *H. halys* microbiota samples. Relative abundance of the three most abundant genera across all individuals. Each bar represents one *H. halys* individual.

Taxonomic classification of microbial communities at the phylum level identified Proteobacteria as the most abundant across all samples and populations (Figure S1). However, compositional differences between the four populations were found at the genus level (Figure 2). The *H. halys* ‐associated microbiota is, in most cases, dominated by a limited number of genera. *Pantoea* was the most abundant genus across SM (Table S1; nonoverwintered: 9/10 samples, overwintered: 4/5) and BS samples (Table S1; nonoverwintered: 6/10 samples, overwintered: 4/4), while *Commensalibacter* was the dominant taxa in most DN samples (Table S1; nonoverwintered: 6/10 samples, overwintered: 3/6). We found high heterogeneity in TN samples, with a similar number of bugs having either *Pantoea*, *Commensalibacter* or *Yokenella* as the most abundant genus (Table S1; 3, 6 and 5 samples out of 16 respectively). These three genera were identified as the most abundant in the four populations (Figure 2). *Yokenella* was the only genus showing complete absence in 37/61 individuals (Tables S1 and S2), whereas *Commensalibacter* (Table S2; overall relative abundance mean: 28.89%, sd: 32.87) and *Pantoea* (Table S2; overall relative abundance mean: 49.58%, sd: 36.14) were present in all individuals.

**FIGURE 3:**
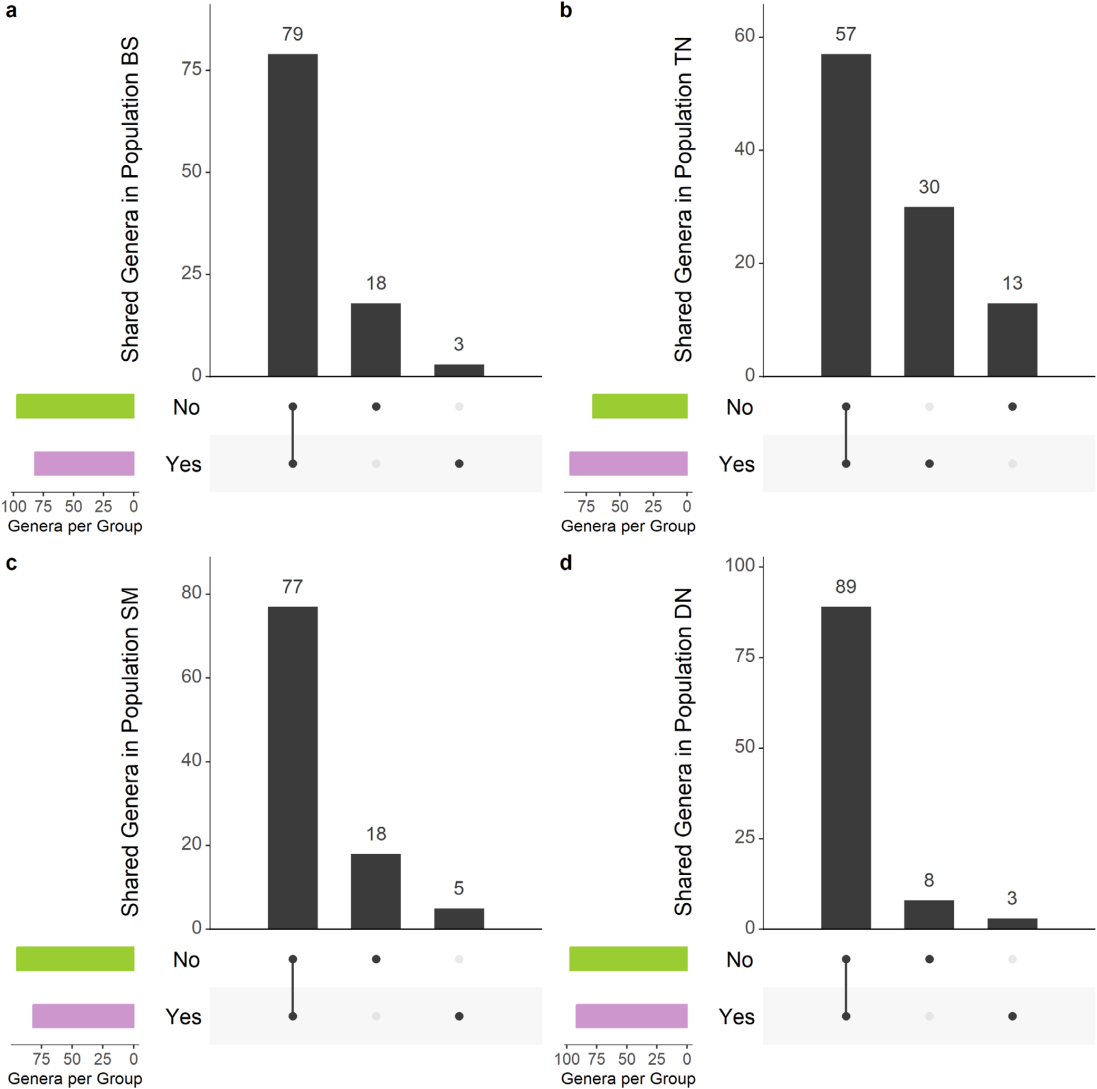
Upset plots depicting relative frequencies (as percentage) of shared genera across the four populations (a, BS; b, TN; c, SM; d, DN) in relation to overwintering. Numbers on top of bars represent the percentage of shared genera with a relative abundance of at least 0.3%. The x‐axis represents the populations, while the y‐axis represents the number of shared genera. ‘No’ for nonoverwintered bugs, ‘Yes’ for overwintered bugs.

**FIGURE 4:**
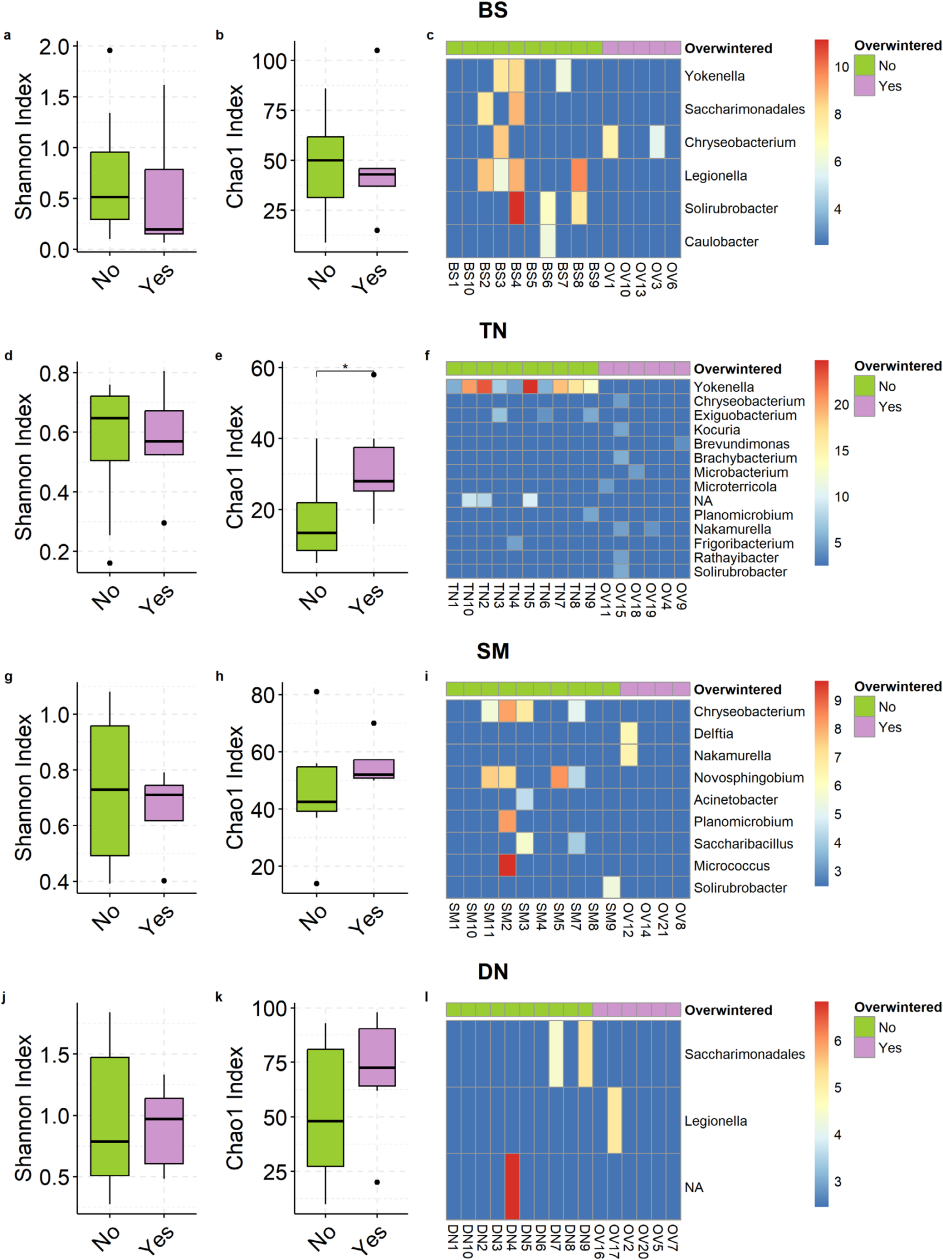
Comparison of alpha diversities of bacterial microbiota in each of four sampled *H. halys* populations at the genus level. (a, b, c) Shannon (a) and Chao1 (b) indices and differentially present ASVs (c) computed for the Besenello (BS) population. (d, e, f) Shannon (d) and Chao1 (e) and differentially present ASVs (f) indices computed for the Trento (TN) population. (g, h, i) Shannon (g) and Chao1 (h) and differentially present ASVs (i) indices computed on the San Michele all'Adige (SM) population. (j, k, l) Shannon (j) and Chao1 (k) indices and differentially present ASVs (l) computed on the Denno (DN) population. In heatmaps (c, f, i, l), the cell coloration varies depending on the z‐score value. Blue indicates the absence of the ASV in the sample, red indicates the highest presence in that sample. *p < 0.05. ‘No’ for nonoverwintered bugs, ‘Yes’ for overwintered bugs.

**FIGURE 5:**
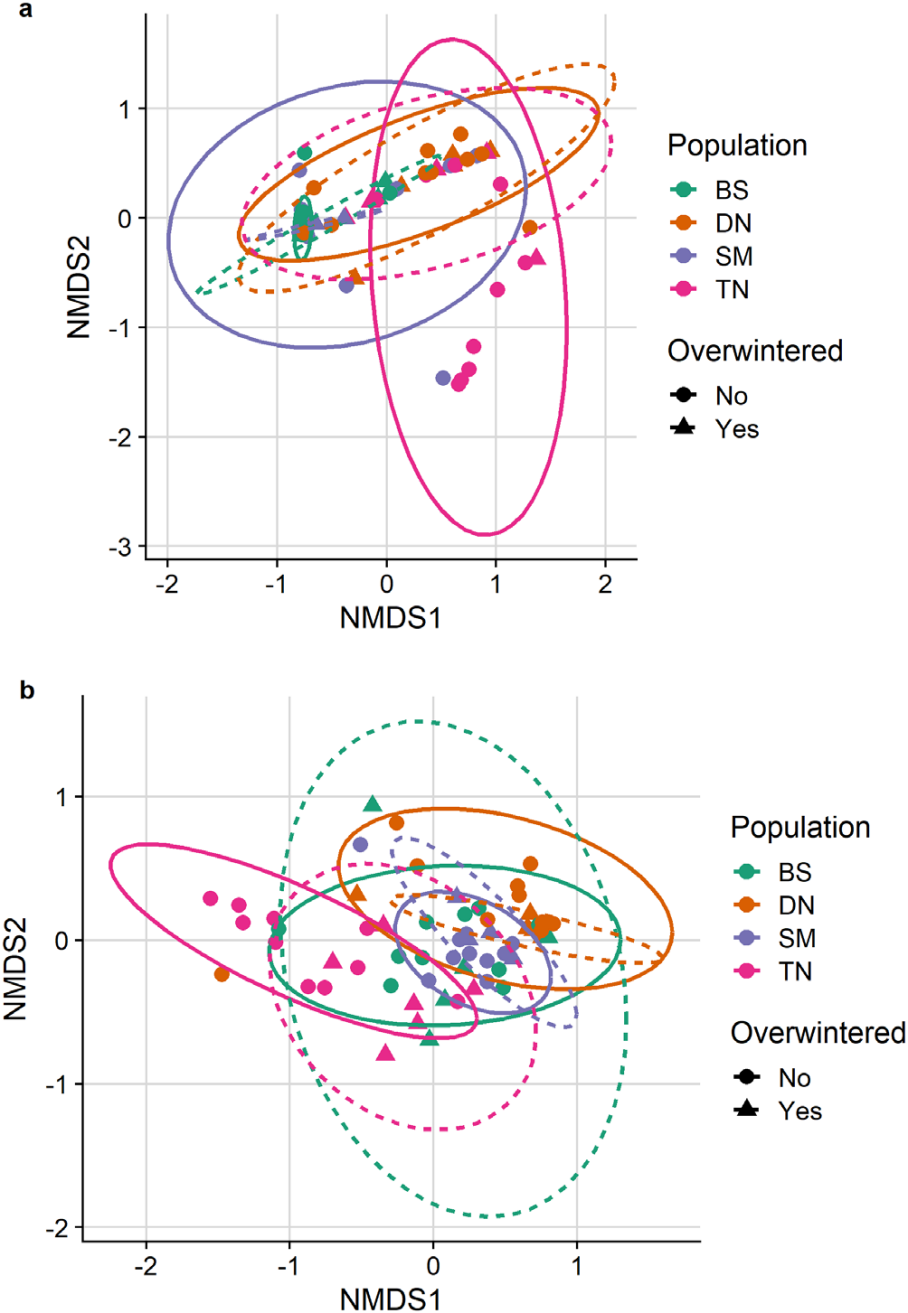
Nonmetric multidimensional scaling (NMDS) plot of sampled *H. halys* microbiota based on Bray–Curtis estimates (a) and Jaccard distance (b).

Most genera were shared among overwintered populations (Figure 3), except TN (Figure 3b), where only 57% of genera were shared between overwintered and nonoverwintered bugs. Alpha diversity (richness; Figure 4) significantly increased in TN overwintered bugs (Figure 4e), but this increase was not observed in the other three populations. Consistently, TN was the only population showing a large effect size of overwintering on the Chao1 index (Table S7). Additionally, TN was the only population showing significant differences in terms of beta diversity between overwintered and nonoverwintered bugs (Figures 5, S3; Table 1; Bray–Curtis: NMDS stress = 0.108, *R* ^2^ = 0.20, *p* ≤ 0.05, Jaccard: NMDS stress = 0.162, *R* ^2^ = 0.19, *p* ≤ 0.01). ANOSIM results corroborated the PERMANOVA results (Table S6; Bray‐Curtis: R^2^ = 0.05, *p* ≤ 0.05, Jaccard: *R* ^2^ = 0.24, *p* ≤ 0.005). The differential abundance analysis at the genus level (Figure 4c,f,i,l) identified a low proportion of genera whose presence significantly differed between overwintered and non‐overwintered bugs (proportion of genera: BS—0.66%, TN—3.91%, DN—0.39%, SM—1.40%). TN showed the highest level of differentiation, primarily attributable to taxa belonging to the genus *Yokenella*, which was abundant in nonoverwintered bugs but absent in overwintered bugs (Figure 4f; Table S4). Similarly, this genus was present in four BS nonoverwintered bugs but not detected in overwintered bugs (Figure 4c). We did not observe significant covariance between Bray–Curtis dissimilarity estimates and geographic distances (Mantel test, *R* = 0.021, *p*: 0.21), but there was a weak significant association between Jaccard dissimilarity estimates and geographic distances (Mantel test, *R* = 0.2, *p* < 0.001).

**TABLE 1:** Results of PERMANOVA analysis based on Bray–Curtis and Jaccard distances and considering interaction and not interaction between population and overwintered factors.

Distance method	Variable	*R* ^2^	*F*	adjusted *p* value
Bray–Curtis	Population	0.275	7.500	0.001 (***)
	Overwintered	0.027	2.174	0.097
	Population*Overwintered	0.050	1.357	0.206
Jaccard	Population	0.207	5.095	0.001 (***)
	Overwintered	0.026	1.946	0.033 (*)
	Population*Overwintered	0.051	1.250	0.135

### 3.2 Detection of Phytopathogens and *Nosema Maddoxi*

Among the screened phytopathogens, we only detected the presence of the genus *Pseudomonas* (52 ASVs) in 45 bugs (TN: 5/10 overwintered, 1/6 nonoverwintered; BS: 9/10, 3/5; SM: 9/10, 4/4 and DN: 8/10, 6/6). Of note, some of these ASVs were assigned at the species level, revealing the presence of *P. cichorii* in one SM overwintered sample with a relative abundance of 0.02%, and *P. tolaasii* in six SM samples (1/4 overwintered, 5/10 nonoverwintered) with a total relative abundance of 0.17%. *Nosema maddoxi* was detected in 13 individuals from two sampling locations: TN (Table S5; 4/10 nonoverwintered, 1/6 overwintered) and BS (Table S5; 6/10 nonoverwintered, 2/5 overwintered).

## 4 Discussion

### 4.1 *Pantoea*, *Commensalibacter* and *Yokenella* are the Most Abundant Genera in *H. halys*

Our characterisation of the whole *H. halys* microbiota showed that *Pantoea* was the most abundant genus overall with high prevalence across populations; this result is in agreement with (Bansal et al. 2014) who identified *P. carbekii* as the primary symbiont of *H. halys* . The high relative abundance and prevalence of this taxon within the bug microbiota, and its prevalence across distant and geographically isolated populations is likely explained by the vertical transmission of microbiota observed in *H. halys* and other Pentatomidae species (Coolen et al. 2024; Gonella et al. 2020; Kikuchi et al. 2012; Prado and Almeida 2009) that promotes early bacterial colonisation of the newly emerged nymphs. More specifically, the mother leaves secretions containing microbes over the egg mass surface (Coolen et al. 2024; Gonella et al. 2020), which are ingested by newly emerged nymphs (Coolen et al. 2024; Gonella et al. 2020). Interestingly, blocking this mechanism by sterilising the egg mass surface causes severe negative effects on reproduction, growth and development of *H. halys* (Taylor et al. 2014).

However, *Pantoea* was not the dominant genus in more than 50% of bugs collected here from two populations: TN (11/16) and DN (9/16). Notably, *Pantoea* was still detected in all the analysed individuals, but at a much lower relative abundance (Table S1). Primary symbionts are considered to be essential for the survival and reproductive success of their hosts and are expected to supplement their host's diet with amino acids or vitamins that are rare or absent in the food source (Ferrari and Vavre 2011). For example, recent studies demonstrated that the genome of *P. carbekii* encodes genes involved in primary metabolic processes (Gonella et al. 2020; Kenyon et al. 2015), whose absence has been shown to negatively affect the development of nymphs and the survival of the following generation (Taylor et al. 2014). However, the relatively low abundance of *Pantoea* in two populations suggests that other genera may also play important roles in *H. hayes* biology. Individuals characterised by low abundances of *Pantoea* were dominated by *Commensalibacter* spp. and/or *Yokenella* spp. The former has also recently been reported by Fluch et al. (2024) for other populations in northern Italy. Bacteria belonging to this genus have been found in other insects, such as honey bees ( *Apis mellifera* L., A. *florea* F. and *A. dorsata* F.), fruit fly ( *D. melanogaster* ), butterflies (*Heliconius* spp.) and the Welwitschia bug (*Probergrothius angolensis* Distant), where it was suggested to be involved in the suppression of proliferation of deleterious microorganisms (Botero et al. 2023).

Unlike *Pantoea* and *Commensalibacter*, which were present in all individuals, albeit at varying abundances, *Yokenella*, the third most abundant genus, was absent in 24/61 individuals. Notably, 16 of these were overwintered bugs, while the remaining five overwintered bugs had very low *Yokenella* abundance (Table S1, Table S2, Table S4). In fact, the population‐level differential analysis between overwintered and nonoverwintered bugs revealed a significant difference in the presence of *Yokenella* in TN and BS populations (Figure 4). The effects of the interaction of *Yokenella* with Pentatomidae species are poorly known, but these bacteria may play an important role in nutrition and/or in detoxification and deactivation of plant chemical defences, as shown in *Nezara viridula* (L.) (Medina et al. 2018), similarly to other Enterobacteriaceae (Gonella et al. 2020), as it was also found in Lepidoptera (*Plutella xylostella* L., Xia et al. 2018). Interestingly, TN bugs were collected in a heavily trafficked urban area near a cornfield, whereas the other sampling locations were situated near apple orchards and/or vineyards in less trafficked areas. Therefore, if functions provided by *Yokenella* hosted by *H. halys* are conserved and similar to those reported for other pentatomids, the observed changes in TN and BS bugs may be linked to dietary shifts. For example, bugs kept in captivity before overwintering were not fed corn, the key crop in the TN sampling location, potentially altering the microbial communities associated with their diet. Additionally, we cannot rule out the possibility that *Yokenella* played a role in the detoxification of pollutants present near sampling locations, particularly in the TN area characterised by heavy road and air traffic (Medina et al. 2018; Xia et al. 2018). Since these pollutants were absent in the cages, the ecological role of *Yokenella* in detoxifying pollutants may have become less critical for the overwintered bugs which, together with diet, could explain its loss or reduction in relative abundance to undetectable levels. However, further populations will need to be studied to test these hypotheses.

### 4.2 Geographically Adjacent Populations Have Diverse Microbiota

Overall, our study showed that *H. halys* ‐associated microbiota varies between sampling sites, as shown by the taxonomic classification of microbial communities (Figure 1) and by the finding that 21% (Jaccard) to 27% (Bray–Curtis) of variation in dissimilarity estimates could be explained by differences across sampled populations (Table 1; Table S3). Consistently, about 40% of identified bacterial genera were not detected across all four populations or were present below the abundance threshold of 0.3% (Figure S2). We also found a weak and significant association between community composition and geographic distance when considering Jaccard dissimilarity estimates (but not Bray–Curtis), indicating that geographic distance is not linked with the abundance of most bacterial taxa, but may affect species composition, particularly of rare taxa. These findings support the hypothesis that *H. halys* ‐associated microbiota harbours a set of abundant bacteria that are most likely important for its survivability (e.g., primary symbionts, see below), and a population of less abundant bacteria (e.g., supported by Chao1 and Jaccard estimates, both of which are sensitive to rare taxa) might be shaped depending on environmental factors such as food availability (Brunetti et al. 2022), usage of pesticides (Syromyatnikov et al. 2020) and/or climate/microclimate conditions (Iltis et al. 2022; Nguyen et al. 2021).

Possible explanations for the significantly different microbial communities hosted by TN bugs compared to the other three populations could be traced back to at least two different processes: variation in diet and anthropogenic disturbance. Since *H. halys* is a polyphagous species, the observed shift in microbial community composition could be due to differences in the composition or relative abundance of available plant species for feeding (Garcia‐Mantrana et al. 2018; Lee et al. 2017; Tang et al. 2012). Similarly, we cannot rule out that variation in microbial diversity observed in TN might be due to anthropogenic disturbance, such as the frequency and composition of chemical treatments, that can influence the *H. halys* microbiota and that are not present in the other sampling locations (Giambò et al. 2021; Juma et al. 2020; Syromyatnikov et al. 2020). We advocate that microbial analyses of *H. halys* (and other insect pests) from the field should be completed for many more populations and coupled with an analysis of the plant species present in the DNA extracted from the bugs and a survey of the vegetation in the collection, as well as other environmental parameters. This would help model and predict the factors responsible for any observed shift in microbial community composition in the sampled insects. Moreover, it is possible that *H. halys* was introduced to Trentino multiple times, with founding populations harbouring distinct microbial communities, which could explain the differences observed among geographically neighbouring populations (Berteloot et al. 2024). To verify this hypothesis, a population genetics study should be conducted at the sampling locations considered in this study to determine the origin of and gene flow between populations; a shotgun approach to microbiota characterisation would also allow the association of genotypes with bacterial strains in the microbial communities.

### 4.3 Overwintering Does Not Affect *Halyomorpha halys* Microbiota

Exposure to harsh conditions such as starvation and cold, which are associated with overwintering for many insects that survive in temperate countries (Vercher et al. 2023), have been shown to induce seasonal shifts in insect microbial communities ( *Diploptera punctata* (Eschscholtz): Ayayee et al. 2020; *G. veletis* : Ferguson et al. 2018; *Hermetia illucens* (L.): Yang et al. 2021). In this study, we aimed to replicate natural overwintering conditions to obtain results as closely aligned with field observations as possible. However, this approach resulted in low survival rates among the overwintering insects, leading to an unbalanced dataset (i.e., 40 nonoverwintered vs. 21 overwintered individuals), which may have biased population‐level comparisons. Consequently, after confirming that parametric test assumptions were not met, we employed nonparametric tests to ensure robust and reliable statistical analyses. Our study showed that for *H. halys* overwintering exerts a very little effect on diversity and composition of its associated microbiota (Figure S4). We found no significant differences in richness (Chao1) or Shannon diversity estimates between nonoverwintered and overwintered populations, with the notable exception of bugs collected in TN. In these, we observed an increase in richness (but not in Shannon diversity) in overwintered bugs. This is likely attributable to differences in microbial composition and richness, since 17% more genera were identified (Figure 2), with one genus, *Yokenella*, showing a significant change in presence (Figure 4f) between nonoverwintered and overwintered bugs. This increase in richness may sound counterintuitive, as it occurred during the period when bugs were in a dormant state. However, the loss of dominant bacteria could create ecological niches that allow other microbial taxa to proliferate, thereby increasing microbial richness. This hypothesis is also supported by the beta diversity results; for example, we found no significant changes in Bray–Curtis dissimilarities, and, although differences were significant when considering Jaccard dissimilarity, effect sizes of overwintering on Jaccard dissimilarities were low‐to‐negligible across all populations (PERMANOVA R^2^ in Table S3; ANOSIM R^2^ in Table S6; Cliff's delta in Table S7). This finding suggests a stable abundance of core taxa dominating the *H. halys* microbiota, even though the composition of bacteria in the microbial community differs between overwintered and nonoverwintered bugs.

The lack of overall effect of overwintering on *H. halys* microbial richness aligns with findings from a recent study by Fluch et al. (2024), which reported no significant differences in alpha and beta diversities of *H. halys* sampled monthly from November to March in the field, thus spanning the overwintering period. These observations align well with the high resilience of some host‐associated microbiota to starvation and diet manipulation documented by Silver et al. (2021) in ground beetles *Anisodactylus similis* LeConte, *Pterostichus serripes* (LeConte) and *Brachinus elongatulus* (Chaudoir). Studies performed in *Periplaneta americana* (L.) demonstrating that starvation did not alter gut microbiota composition or alpha diversity led Tinker and Ottesen (2016) to hypothesise that cockroaches may possess mechanisms for maintaining stable host–microbiota relationships despite nutritional stress. Maintenance of its microbiota during overwintering might provide *H. halys* with the obvious advantage of being able to emerge with a fully functioning microbiota. Maintenance of host‐associated microbial communities during overwintering could be indirectly prompted by the high dominance observed in microbial communities before overwintering (resulting in a similarly high dominance for the same taxa after diapause) and/or the presence of adaptation mechanisms buffering against changes during overwintering‐associated stresses. Aggregation during overwintering may also play a role in maintaining microbiota stability in *H. halys* . The close proximity of individuals might facilitate microbial exchange, potentially via physical contact or faecal transfer, promoting shared microbiota stability across the group (Qin et al. 2022; Schmidt and Engel 2021). While these mechanisms remain speculative, they offer intriguing directions for future research on the interplay between evolution, behaviour and microbiome resilience. Notably, a stable host–microbiota interaction is probably maintained in this species through vertical transmission (Gonella et al. 2020; Martinez‐Sañudo et al. 2018). Therefore, *H. halys* may have evolved and selected complementary mechanisms, vertical transmission for initial microbial colonisation and stability mechanisms for preserving microbial composition during overwintering, to sustain a stable host–microbiota association across both developmental and environmental challenges.

### 4.4 Implications for the Control of *Halyomorpha halys* and Other Insect Pests

The stability of microbiota composition during harsh overwintering conditions is relevant to the efficacy of various biological control strategies, such as SIT. This technique is a validated biological pest control strategy based on mass rearing, sterilisation and inundative releases of predominantly male sterile insects, with male sterility induced using bacteria (Enkerlin W et al. 2017; Vreysen et al. 2000) or by irradiation with gamma or X‐rays (Klassen et al. 2021). It has been suggested that SIT could be used to eradicate or suppress *H. halys* when they colonise a new area (Welsh et al. 2017). However, in order to successfully apply this technique, the bugs must be mass‐reared in laboratory conditions while maintaining their competitiveness with wild individuals, an approach which has not been perfected for various hemimetabolous insects (Lance and McInnis 2021), including *H. halys* (Lance and McInnis 2021; Medal et al. 2012; Nguyen et al. 2021; Suckling et al. 2019a; Welsh et al. 2017). Therefore, an alternative approach has been suggested, which involves mass‐capturing wild bugs from areas of high density, inducing overwintering, sterilising them after reproductive diapause, then releasing them in the planned area (Roselli et al. 2023; Suckling et al. 2019a). For this reason, the screening process for better understanding of the effect of overwintering on *H. halys* microbiota, with special reference to the potential plant pathogens carried by *H. halys* , is of utmost importance to minimise the likelihood of their accidental introduction. However, unlike other organisms used in SIT programmes such as male moths or Tetriphidae (Lance and McInnis 2021), sterilised adult male (and female) *H. halys* continue to feed on plant hosts inducing wounds and likely transmitting phytopathogens. In such cases, it is important not only to focus on midgut microbiota but also to consider the external surface and other internal organs. For example, it is important to investigate plant pathogens accidentally carried on the exoskeleton that can access the plant through wounds, such as *Pseudomonas syringae* (Van Hall) (Orlovskis et al. 2015; Tian et al. 2024), pathogens present in the salivary glands that can be transmitted through the saliva, such as phytoplasmas (Christensen et al. 2005), or pathogens known to infect different organs, such as *N. maddoxi* that can infect haemolymph, fat bodies and reproductive organs (Rivers et al. 2022). Therefore, whole‐body metataxonomics, although increasing the risk of not detecting underrepresented microorganisms, offers conservative detection of microbes localised across tissues and on the exoskeleton surface, including potential pathogens.

Insect vectors can transmit multiple species of phytoplasma (Lee et al. 1998) and assessing the presence of these pathogens in polyphagous pests such as *H. halys* is important for agricultural pest management. For example, in many countries, the productive output of apple trees and grapevines is threatened by *H. halys* , and these same crops are subject to diseases caused by phytoplasmas (i.e., apple proliferation phytoplasma, grapevine flavescence dorée and grapevine bois noir). *H. halys* has been shown to host witches' broom phytoplasma, a threat for princess trees ( *Paulownia tomentosa* ) and other plants (Gao et al. 2008; Jones and Lambdin 2009). However, consistent with previous reports (Hoebeke and Carter 2003; Tassini and Mifsud 2019), we did not detect phytoplasmas in our samples of *H. halys* , suggesting that phytoplasmas are unlikely to be transmitted by *H. halys* in this area.

Instead, we detected *Pseudomonas* genus in 45/61 (74%) *H. halys* , and more specifically in the study area, the species *P. cichorii* and *P. tolaasii* . *P. cichorii* is a phytopathogen with a wide host range, which causes leaf blighting and spotting (Trantas et al. 2013), while *P. tolaasii* occasionally causes bacterial blotch on cultivated mushrooms (Liu et al. 2022). Since all bugs infected with these two *Pseudomonas* spp. originated from a single location (SM), we suggest these pathogens might be location‐specific. This result highlights the importance of environmental factors in shaping the *H. halys* ‐associated microbiota. Although our findings suggest that *H. halys* is unlikely to act as a primary vector for these bacteria due to their relatively low prevalence, even sporadic occurrences could influence plant disease dynamics, particularly if *H. halys* contributes to localised bacterial spread via feeding wounds and excrements (Orlovskis et al. 2015; Tian et al. 2024). Further studies should assess whether *H. halys* can facilitate the mechanical transmission of these phytopathogens and under what conditions they might contribute to plant infections.

The consistent prevalence of *N. maddoxi* in only two neighbouring sampling sites, TN and BS, suggests that the presence of this parasite might be location‐specific. Although this result seems to be in contrast with previous findings, we sampled the bugs as soon as they emerged from their diapause and not in the field during spring as in Kereselidze et al. (2020) and Preston et al. (2020a). It is possible that when *H. halys* bugs aggregate prior to and during the overwintering period, *N. maddoxi* propagates from the few infected individuals by horizontal transfer (Preston et al. 2020a). By spring, when overwintered *H. halys* survivors begin dispersing, many of them may be infected with *N. maddoxi*, which could decline in the population over the summer and reappear the following season, as shown in Preston et al. (2020a). This pattern may explain why we found more infected bugs before overwintering compared to emergence after diapause and why previous studies reported higher infection rates in spring than in winter. Notably, approximately 70% of the bugs in our study did not survive the overwintering period, a result consistent with the literature (e.g., Costi et al. 2017; Lowenstein and Walton 2018). However, the detection of infected individuals in both TN and BS confirms that *N. maddoxi* is retained in the population and could be transmitted in the following season. Hajek et al. (2023) corroborated this idea by finding that *N. maddoxi* was more prevalent in dead *H. halys* during overwintering, suggesting that the microsporidian might contribute to overwintering mortality, but survivors can experience increasing infection levels by the spring.

The release of *N. maddoxi*‐positive individuals entails potential risks that must be carefully evaluated. Although *N. maddoxi* has been suggested as a natural population suppressor by contributing to overwintering mortality (Hajek et al. 2023), it could also spill over to nontarget pentatomids, such as *Chinavia hilaris* (Hajek et al. 2018), or alter microbial community dynamics in invaded agroecosystems. Furthermore, if *N. maddoxi* negatively affects host fitness, it could reduce the effectiveness of released sterile individuals in mating competition, potentially diminishing SIT success. Therefore, screening for *N. maddoxi* presence in wild‐captured individuals prior to sterilisation would be necessary to minimise unintended ecological consequences.

Our findings suggest that microbial screening could be performed on a subsample of collected bugs prior to overwintering, given the similarity between the microbiota of freshly captured and overwintered individuals. However, careful risk assessment is essential before incorporating *N. maddoxi*‐positive individuals into control strategies. Future research should include metataxonomy to evaluate the ecological implications of using infected insects in SIT programmes, including potential interactions with native species and broader agroecosystem impacts.

## Author Contributions

**Riccardo Piccinno:** writing – original draft, methodology, data curation, formal analysis, investigation, writing – review and editing, visualization. **Giulio Galla:** investigation, formal analysis, supervision, methodology, writing – original draft, writing – review and editing, visualization, data curation. **Gerardo Roselli:** methodology, writing – review and editing. **Mirco Rodeghiero:** writing – review and editing. **Valerio Mazzoni:** resources, writing – review and editing. **Lloyd Stringer:** conceptualization, writing – review and editing. **Heidi Christine Hauffe:** writing – original draft, writing – review and editing, supervision, resources, validation, funding acquisition. **Gianfranco Anfora:** conceptualization, writing – review and editing, resources, funding acquisition. **Omar Rota‐Stabelli:** conceptualization, investigation, writing – original draft, writing – review and editing, supervision, resources, project administration, funding acquisition.

## Ethics Statement

The authors have nothing to report.

## Conflicts of Interest

The authors declare no conflicts of interest.

## Supporting information


**Data S1.** Supporting Figure.

**Data S2.** Supporting Tables.

**Data S3.** Supporting Information.

## Data Availability

The data that supports the findings of this study are available in the supplementary material of this article.
