# Supplementary material for: Overwintering Does Not Affect Microbiota Diversity in Halyomorpha halys : Implications for Its Ecology and Management
Source: Environ Microbiol Rep. 2025 Jun 10;17(3):e70116. doi: 10.1111/1758-2229.70116 (PMC12149765; doi:10.1111/1758-2229.70116)
Supplement: Supplementary file 1 — Data S1. Supporting Figure. [file EMI4-17-e70116-s003.pdf]

Supplementary Figures

Phylum-level population abundance

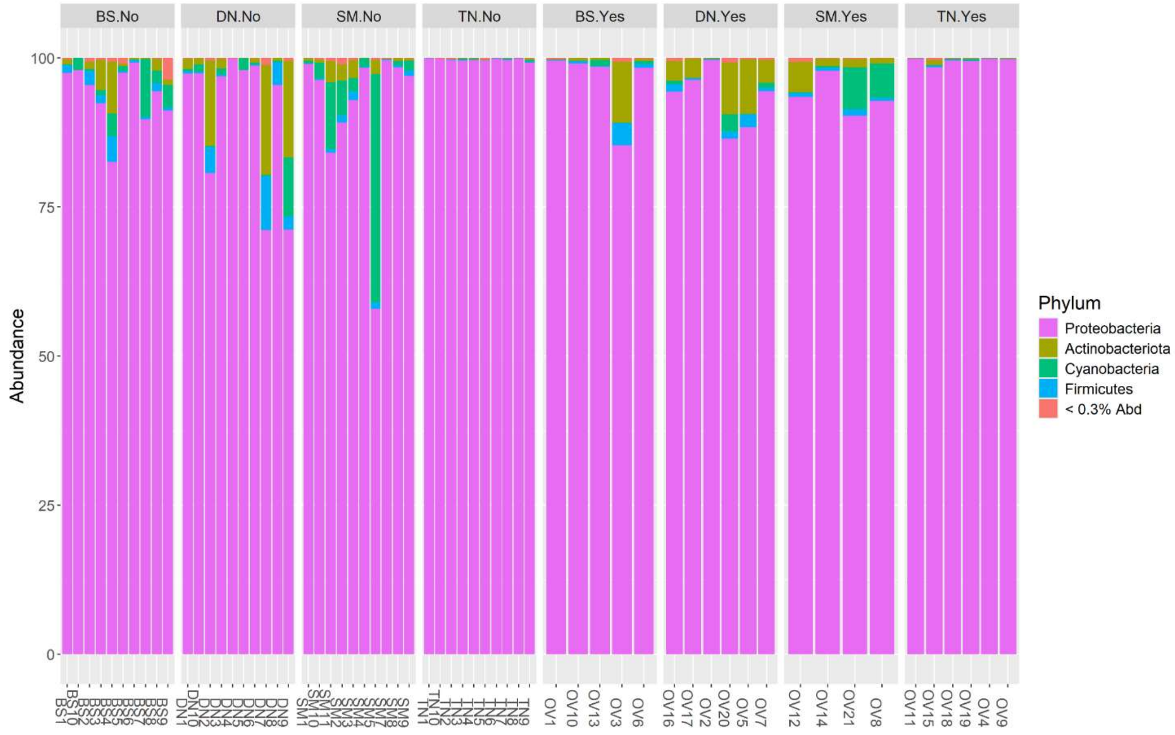

Fig. S1 Comparison of whole *H. halys* microbiota diversity and composition between sampling locations at the phylum level. Relative abundance plot of the four most present phyla identified (Abd < 0.3%: all phyla with a sample relative abundance < 0.3%). Each bar represents a *H. halys* individual. "No" for non-overwintered bugs, "Yes" for overwintered bugs.

Genus-level samples aggregated by population and overwintering

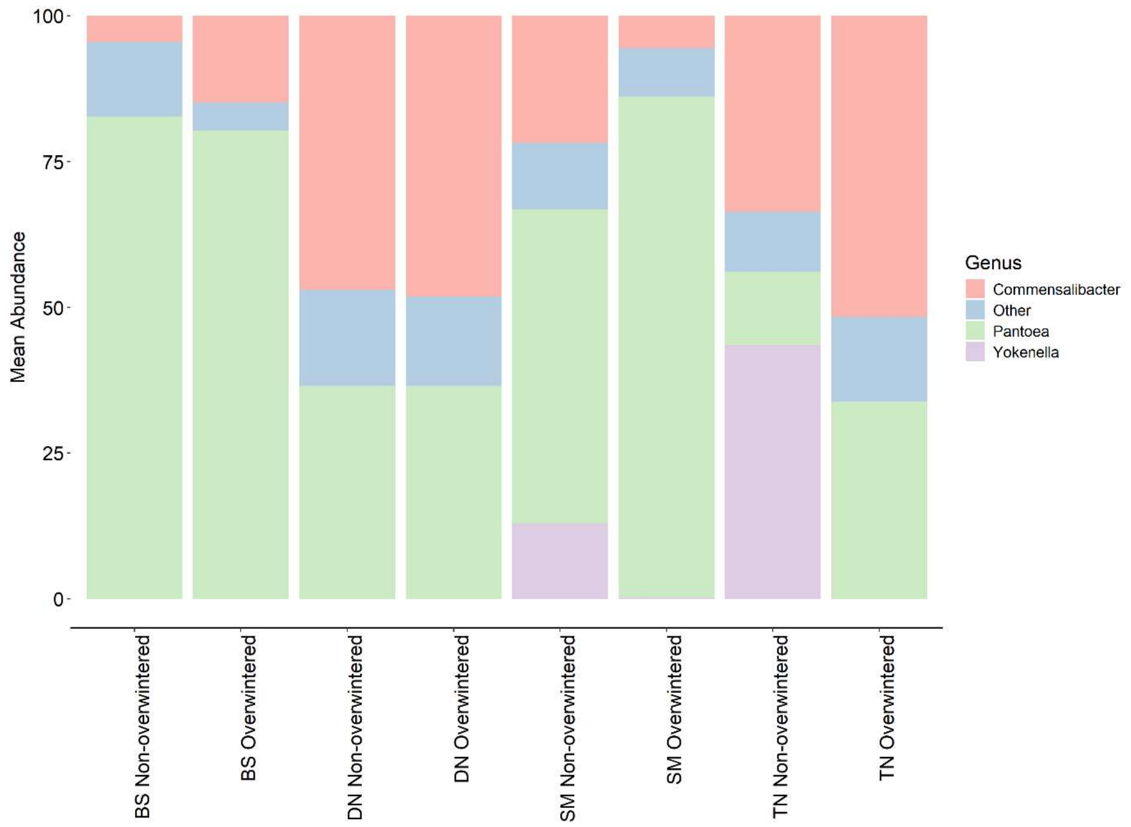

Fig. S2 Abundance plot showing bacterial genera in all *H. halys* microbiota samples. The relative abundance of the three most abundant genera is displayed across all individuals. Samples were grouped based on their population and overwintering status. Each bar represents the mean relative abundance for each combination of these two variables.

Population analysis

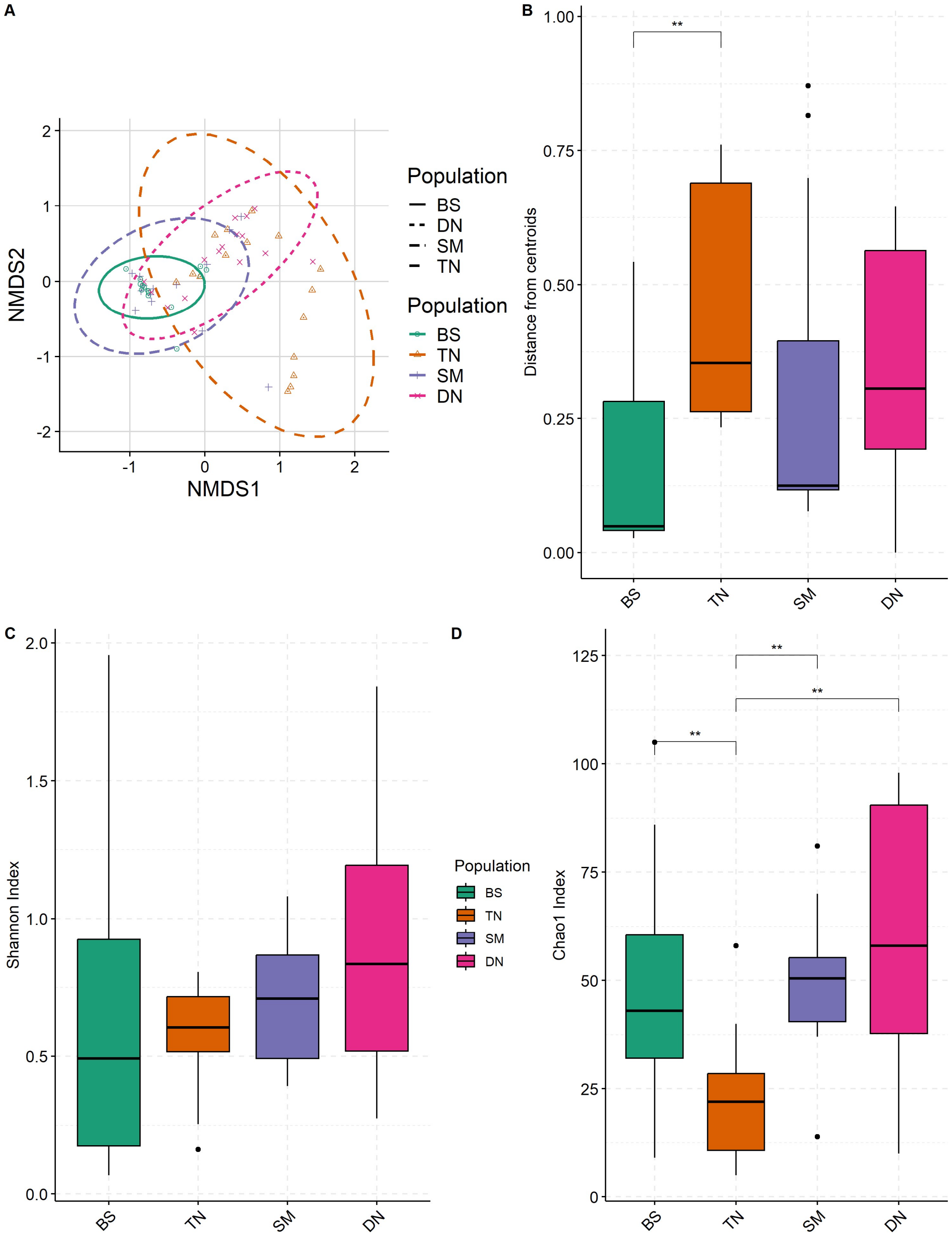

Fig. S3 Comparison of whole *H. halys* microbiota diversity and composition between sampling locations at the genus level. (A) Nonmetric multidimensional scaling (NMDS) plot of sampled *H. halys* microbiota. Permanova based on dissimilarities was also estimated (R^2 = 0.17745; P = 0.002). (B)

Distances from centroids computed using Bray-Curtis method. (C, D) Alpha diversity plots of Shannon (C) and Chao1 (D) indices at the genus level. ** $p < 0.01$.

Overwintering analysis

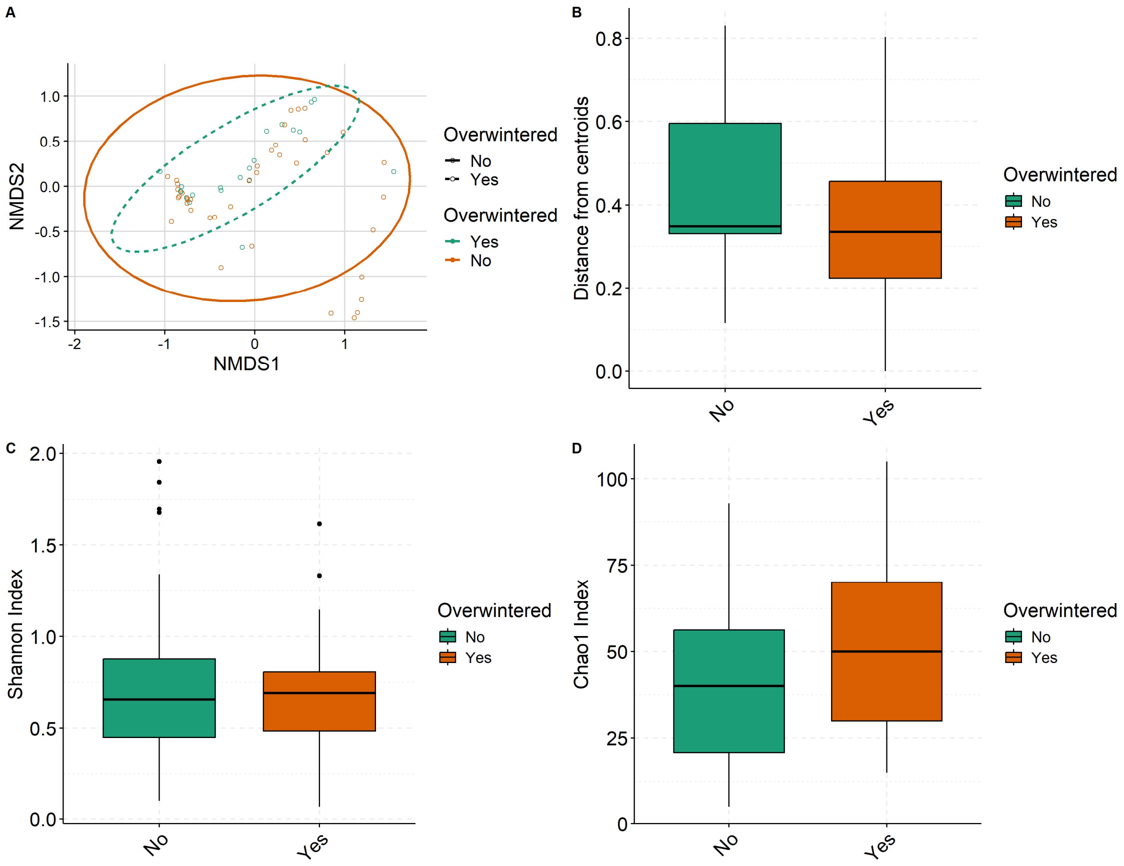

Fig. S4 Comparison of microbiota composition and diversity between not overwintered and overwintered *H. halys* at the genus level. (A) NMDS plot of sampled *H. halys*. Permanova based on dissimilarities was also estimated ($R^2 = 0.0218$, $p = 0.254$). (B) Distances from centroids computed using Bray-Curtis method. (C, D) Alpha diversity plots of Shannon (C) and Chao1 (D) indices at the genus level.
