# Supplementary material for: Overwintering Does Not Affect Microbiota Diversity in Halyomorpha halys : Implications for Its Ecology and Management
Source: Environ Microbiol Rep. 2025 Jun 10;17(3):e70116. doi: 10.1111/1758-2229.70116 (PMC12149765; doi:10.1111/1758-2229.70116)
Supplement: Supplementary file 3 — Data S3. Supporting Information. [file EMI4-17-e70116-s001.pdf]

Supplementary Information

DNA extraction protocol

This protocol was optimized according to the manufacturer's instructions.

The first part of the protocol (steps 2-9) was taken from the "Protocol: Pretreatment for Gram-Positive Bacteria" (pp. 45-46 of QIAGEN DNeasy Blood and Tissue kit manual). The last steps (10-13) were taken from the "Animal Tissue (Spin-Column Protocol)" (p. 30).

1. Place a bug in a 2 ml DNA-DNAse free tube containing a 5 mm sterile stainless steel bead (QIAGEN). Flash freeze the sample in liquid nitrogen for a minimum of 30 seconds and then grind it using a Tissuelyser II (QIAGEN) for 1 minute at 30 Hz, or until the bug inside is completely powdered.
2. Add 320 µL of the lysis buffer (20 mM Tris-Cl pH 8, 2mM sodium EDTA, 1.2% Triton® X-100) to the sample.
3. Vortex for 20 seconds to obtain the lysis mixture, then spin for 10 seconds.
4. Pipette 162 µL of the pellet of the lysis mixture in a new 2 ml DNA/DNAse-free tube and store the remaining mixture at -80 for future uses. Depending on the mixture composition, it might be needed to cut the pipette tip with a sterile DNA/DNAse-free scalpel to allow the pipetting of the mixture.
5. Add 18 µL of 200 mg/mL lysozyme to the mixture (to reach the final concentration of lysozyme of 20 mg/ml) and mix thoroughly.
6. Incubate for at least 30 min at 37°C. Then spin for 10 seconds.
7. Add 25 µL proteinase K and 200 µL Buffer AL (without ethanol) and vortex.

8. Incubate at 56°C for 30 minutes. Then spin for 10 seconds.
9. Add 200 µL ethanol (96-100%) to the sample and vortex. Then spin for 10 seconds.
10. Pipet the mixture into the DNeasy Mini spin column placed in a 2 ml collection tube. Centrifuge at 8000 rpm for 1 minute. Discard the flow-through and the collection tube.
11. Place the DNeasy Mini spin column in a new 2 ml collection tube, add 500 µL Buffer AW2, centrifuge for 1 minute at 8000 rpm, then centrifuge for 2 minutes at 14000 rpm to dry the DNeasy membrane. Discard the flow-through and the collection tube.
12. Place the DNeasy Mini spin column in a clean 1.5 ml microcentrifuge tube, and pipet 50 µL Buffer AE directly onto the DNeasy membrane.
13. Repeat 12 another time.

Eluted DNA was conserved at -18°C.
